# Wireworms’ Management: An Overview of the Existing Methods, with Particular Regards to *Agriotes* spp. (Coleoptera: Elateridae)

**DOI:** 10.3390/insects4010117

**Published:** 2013-01-25

**Authors:** Fanny Barsics, Eric Haubruge, François J. Verheggen

**Affiliations:** Department of Functional and Evolutionary Entomology, Gembloux Agro-Bio Tech, University of Liege. 2, Passage des Déportés, 5030 Gembloux, Belgium; E-Mails: e.haubruge@ulg.ac.be (E.H.); fverheggen@ulg.ac.be (F.J.V.)

**Keywords:** wireworms, click beetles, *Agriotes*, integrated pest management

## Abstract

Wireworms (Coleoptera: Elateridae) are important soil dwelling pests worldwide causing yield losses in many crops. The progressive restrictions in the matter of efficient synthetic chemicals for health and environmental care brought out the need for alternative management techniques. This paper summarizes the main potential tools that have been studied up to now and that could be applied together in integrated pest management systems and suggests guidelines for future research.

## 1. Introduction

Wireworms are the larvae of click beetles (Coleoptera: Elateridae). They consist of more than 9,000 species distributed worldwide, [1] and some are important pests of a wide variety of crops, such as potato, cereals, carrot, sugar beet, sugarcane and soft fruits (e.g., [2,3,4,5,6]). In Europe, damages due to wireworm infestation are mainly attributed to the genus *Agriotes* Eschscholtz, as witnessed by the numerous studies aiming at their management. It comprises more than 200 species worldwide, including more than 100 in the Palearctic region [1]. Different recent sampling plans set in all of Europe (pheromone traps, molecular identification of collected larvae) allowed a reliable mapping of species, showing that *Agriotes* communities were widely distributed across the continent [7,8,9,10,11,12,13]. Their distribution in North America is also well described [14,15,16]. The genus *Melanotus* Eschscholtz is well studied in Japan (*Melanotus okinawensis* Ohira) and in the USA (*Melanotus communis* (Gyll.)) (e.g., [17,18,19,20]). The “Pacific coast” wireworm, *Limonius canus* Leconte, is responsible, with other species of the genus, for crop damage alongside the western coast of North America (e.g., [21,22,23]). Other genera important for their impact on agriculture are *Athous* Eschscholtz, *Conoderus* Eschscholtz, *Ctenicera* Latreille and *Hypolithus* Eschscholtz (e.g., [16,17,18,24,25,26,27,28,29,30,31,32,33]). The main effects of wireworm feeding on neck and belowground plant organs are seedling mortality [34] and all implied yield losses. Damage to potato tubers largely consists of small holes, narrow tunnels, a couple or more millimetres deep, and scarring to the periderm, which can significantly reduce tuber quality [6,35]. 

Despite the numerous studies conducted to improve their management, wireworms remain important pests. In the UK, lindane and aldrin remained the mainstay of wireworm control in potato crops, until well into the 1990s. Their apparent effectiveness even stifled research on alternative control strategies for thirty years (1960 to 1990) [36]. Their withdrawal from registration for environmental and health concerns gave rise to the need of alternative control methods [36]. Soil-incorporated organophosphorous and carbamate insecticides replaced organochlorines, but observed control levels were overall variable [37,38]. Due to the same adverse secondary effects, including long persistence in the environment (insecticidal wireworm treatment can be linked to dramatic effects on non-target species, as shown recently for fonofos [39]) and human health concerns, they were progressively withdrawn from the markets, and those remaining may soon be phased out, even in North America [17,40]. Multiple alternatives to broadcast application of pesticides have been examined worldwide. Their implementation in Integrated Pest Management (IPM) systems should provide good results, but demonstrations of such synergistic interactions are still rare. This paper summarizes the key data that should be considered and the IPM strategies that could be used against wireworms, with particular regards to *Agriotes* spp. in Europe and guidelines for future research.

## 2. Species Identification, Lifecycle and Key Moments for Crop Protection

The key moments for predicting the implementation of IPM strategies are, firstly, adult population emergence (before oviposition and/or field cultivation), in order to prevent population settling or reinforcements and to enhance eggs and first instars mortality. Secondly, the periods during which the larvae supposedly feed actively are important, in regard to the timing of the cultivation of sensitive crops [41]. It is therefore crucial for crop protection to know the species distribution and the features of their lifecycles that help to spot the right timing for control measures.

Adults of all agronomically important species have been well described [1,42,43,44,45,46,47,48]. Although many tools are available for the identification of *Agriotes* wireworms, based on morphology [8,49,50,51,52,53,54] or on genetic footprints [8,55,56], *in situ* direct identification remains an issue. In most cases, determining if individuals belong to a species is more difficult for larvae than for click beetles, sometimes suggesting doubts as to their actual separation [57], but molecular advances have rendered possible community description at field scale [55]. For both click beetles and wireworms, an identification key (morphological, including molecular validation, as was done by Pic *et al.* (2008) for France [8]) that is valid for all regions concerned (mainly Europe) should be prepared that is suitable for use by a large group of interested people [57]. Very recent molecular research suggested the possibility of cryptic wireworm species or misidentifications on *Hypnoidus bicolor* Eschscholtz populations in Canada, highlighting the necessity to consider that approach in identification [58].

Reliable information concerning the biology of the different species can be obtained by studying concurrently their entire cycle under laboratory conditions (rearing chambers, constant temperatures), in rearing cages close to natural conditions and in open fields [57], as reported in several studies [41,59,60]. Their study across different regions is interesting, but a better overall understanding can result from the comparison of development cycles in different environments [61]. Good biological information is available for the following species: *A. ustulatus* Schäller [59,60,62,63], *A. sordidus* Illiger [41,61], *A. brevis* Candeze [64,65] and *A. litigiosus* (information only available for *tauricus* [66]). Insufficient information is available for *A. obscurus* L. [2,34,67,68,69,70,71,72,73,74,75,76], *A. sputator* L. [66,67,69,75] *and A. lineatus* L. [67,72,73]. Information about *A. proximus* Schwarz and *A. rufipalpis* Brullé is almost inexistent and should be completed following the methods used for species whose biology has already been described [57].

Click beetles are characterized by a multi-year lifecycle that differs dramatically between species. They can be divided into two main groups. The first group is constituted of species with adults that do not overwinter, live a few days and lay eggs a few days after swarming (*A. ustulatus*, *A. litigiosus*). The second one assembles species with adults that overwinter and live for months. These lay eggs for a long period after adult hardening (*A. sordidus*, *A. brevis*, *A. lineatus*, *A. sputator*, *A. obscurus*, *A. rufipalpis* and *A. proximus*) [57]. Adults emerge from the soil in spring. *A. sordidus* usually emerges from late March to early April [41]. Females of *A. obscurus L., A. sputator L.* and *A. sordidus* lay their eggs in May or June, singly or in clusters, below the soil surface [2,41,76], while the oviposition peak of *A. ustulatus* occurs in July and early August [59]. Oviposition would be reduced on arable land compared with grass [75], where comparatively more wireworms survive the early instars [77], the eggs benefiting from the protection against desiccation. Conditions in grasslands are so stable that wireworms may remain in the top 12 cm of soil during their whole development [2]. The monitoring of wireworm survival in different rotations (continuous rotation, very short interruption between different crops, discontinuous rotation, long periods with bare soil between crops, meadow) confirms that soil with continuous vegetation cover allows more wireworms to survive [78]. The embryonic development is inversely related to temperature [41,60]: *A. ustulatus* eggs hatch in 13 days when maintained at 29 °C and in 45 days when maintained at 15 °C [60], while in the U.K, under agronomic conditions, *Agriotes* spp. eggs only hatch after 4–6 weeks [2,79]. Temperature also influences wireworm movement: the speed of *A. obscurus-lineatus* increases linearly from 8 to 19 °C in lab conditions, an important factor for behavioral experiments [80]. *Agriotes* larvae rarely disperse between crops or move far in the soil, putatively as long as local food supply is sufficient [33,81]. Resistance to starvation increases with the age of wireworms: the last instars of *A. sordidus* can survive up to one year without food at 20 °C [41]. *Agriotes* wireworms are predominantly phytophagous, with an influence of the type of vegetable food on growth rates [76]. The youngest larvae need live vegetable material to grow [41,60,76]. Besides crops and weeds, *Agriotes* spp. also feed on animal prey. Traugott *et al.* (2008), using naturally occurring stable isotope signatures, highlighted intraspecific trophic plasticity by showing that 10% of a population of *A. obscurus* fed primarily on animal prey [82]. They may even feed on congeners: cannibalistic interactions have been reported, where larval density is high and food is scarce [60,72]. Some studies report on soil organic matter as a possible alternative food source [72,83,84], but there is no proof that wireworms can survive or grow on non-live vegetable tissues only. Recent work proved that high instars of *A. obscurus* predominantly fed on plant material (only 10% on soil organic matter, 95% confidence limits: 0–20%) [82]. In a study based on the isotopic signatures of organisms according to their trophic levels, wireworms kept 128 days without food in moist garden soil didn’t shift their isotopic signatures towards the one of soil, indicating no or only negligible feeding on soil organic matter [85], which confirms observations of Evans and Gough (1942). With PCR-based detection of plant DNA in soil-living insects, such as *Agriotes* larvae [86], it is possible to examine whether general patterns exist as to their dietary choices. Although it is not yet possible to determine the amount of plant tissue per plant species consumed, it was shown that they seem to depreciate decayed plants, while no significant consumption of soil organic matter has been detected with this technique up to now [87]. Living plant material would thus be by far the main food source of *Agriotes* wireworms all along their development, and soil organic matter cannot replace it. 

According to the species, latitude, temperature and food availability during larval development, wireworms may pass through a variable number of instars. *A. sordidus* goes through 8 to 13 instars, depending essentially on the centigrade degree day accumulation (CDDA) since egg hatching. It also influences the number of years needed to complete the cycle and is strongly related to geographical location [41,60,61,88]. *Agriotes* wireworms in the U.K. (mainly *A. obscurus*, *A. lineatus* and *A. sputator*) develop up to four or five years before pupating [2]. In contrast, *A. sordidus* is able to complete its whole lifecycle in 24 months, over two calendar years, provided that food is available and that 4,000 DD are accumulated during the first year of larval development. The cycle can last longer if no food is available for long periods. For some individuals maintained at 29 °C, three months can be enough to complete development from egg to adult [41]. Each larval instar passes through three phases: mandible hardening and darkening, feeding and pre-molting. The intense feeding phase associated with plant damage can last less than 20% (*A.ustulatus*), 25% (*A.sordidus*) or 29% (*A. obscurus*) of the whole development time [41,60,76]. Wireworms generally have two intense activity periods that may result in significant crop damage over one calendar year, according to suitable temperature and moisture conditions in the different soil layers. They occur from March to May and from September to October [34]. The depth at which *A. ustulatus* larvae can be found depends mostly on late autumn, winter and early spring temperatures and on soil moisture during the rest of the year. It can be as deep as 60 cm in the winter, while in spring, provided that soil temperatures are above 10 °C and there is suitable soil moisture content, most of the larvae are in the upper 20 cm of the soil [60]. In between sensitive periods, wireworms perform vertical migration related to soil temperature, soil moisture and soil type [34,60,89], the preferred range of soil moisture being related to soil type, especially to the permanent wilting point and the soil moisture tension [89]. Evaluating the soil conditions during the critical phases of the cycle is of importance, as it shows the suitability of young larvae. High moisture and live roots in the upper layer of the soil in late May-June may cause severe attacks by *A. sordidus* the following autumn and subsequent years if nothing is undertaken to control the population [41]. The first crop threatening instars occur in the root layer sooner or later according to the duration of the lifecycle. *A. obscurus* starts damaging during their first spring, a year after eggs have hatched. They get increasingly harmful season after season [34]. *A. sordidus* wireworms develop faster and reach a damageable size only three to four months after they hatch. They may damage any sensitive crop planted in late summer or early autumn [41]. In some regions, especially with mild climates, impacts on crops may go on all year long, even in the winter [34]. The length of 10mm associated with yield losses is reached between the fifth and sixth instar for both *A. sordidus* and *A. lineatus* wireworms, whilst the latter need two years to acquire it (two instars per year) [34,41]. Most of the last instar larvae transform into pupae and adults in the summer. They pupate in the upper 30 cm of the soil and remain inside cells until the subsequent spring. Soil tillage can however induce cell breakage and adult emergence and exposure before spring [41]. 

## 3. Available Data on the Chemical Ecology of Wireworms

The number of studies reporting on the orientation of soil insects in response to chemical cues released by roots has gradually increased, and the location of the latter by random insect movement is now seldom reported [90]. To our knowledge, the first evidence of such response in wireworms was reported by Thorpe *et al.* (1946). The authors demonstrated elicitation of the biting response when wireworms (*A. lineatus, A. obscurus and A. sputator*) encountered plant juices or solutions containing either one or more of a number of carbohydrate, fatty or protein substances and certain common plant sugars (glucose, fructose, sucrose, galactose, maltose and stachyose). They all induced a biting response threshold progressively lowered with starvation, up to seven days. Substances eliciting orientation were also highlighted (aspartic acid, asparagine, malic acid, succinic acid, glutamine and glutamic acid). The sensitivity for the orientation response tends to vary with the nutritional state, as well as with season and time in relation to molt, as it does for biting [91]. The perception of natural chemicals associated with the host plant will vary accordingly to physiological particularities of the plant, which has an impact on the host-plant choice at the individual scale. For example, *A. obscurus* wireworms are able to avoid high glycoalkaloid (mainly α-solanine and α-chaconine) and low sugar concentrations and also solanine and chaconine, compounds found in potatoes [92], even if these are not the only ones responsible for varietal susceptibility [6]. Wireworms are able to orient towards carbon dioxide by klinotaxis [93]. *Ctenicera destructor* responds to concentration differences of 0.002 mmol mol^−1^ [24]. Carbon dioxide is perceived by clusters of sensilla on the labial and maxillary palps (observed on *Limonius californicus*, *A. obscurus* and *A. lineatus*) [94]. Other species probably perceive CO_2_ this way, as hypothesized for *Limonius canus* [95]. It remains unclear whether other volatile chemicals emitted by food baits or germinating seeds affect wireworm movements [24], and this question obviously merits additional research efforts [95], keeping in mind that physiological differences occur between species [96].

Johnson and Gregory (2006) put forward a model of attraction/location of root feeding insects, including wireworms, towards root systems and based on three important phases [90]. Insects first move at random, any orientation being under the influence of heterogeneity in soil architecture: they follow the lines of least resistance [91]. When they perceive general (non-specific) semiochemical(s), such as CO_2_, the random movement is biased. The stronger vertical gradients (between the air and the upper soil) and the high density of roots question CO_2_ efficiency as a local host-locator, particularly for specialized herbivores in mixed plant communities [90]. The rhizosphere is favorable for volatile-mediated communication, since volatiles are more likely to accumulate and reach their activity threshold than in the wind-exposed above-ground environment [97]. The perception of host specific semiochemical(s), such as volatiles, would therefore elicit recognition of different host species [90]. This step might explain the preference of *A. obscurus* and *A. haemorrhoidalis* for two nutrient-rich grassland plant species next to two nutrient-poor ones, observed by Hemerik *et al.* (2003) [33]. In the belowground interactions, it should be noted that there is a trend for low molecular weight compounds (e.g., alcohols, esters, aldehydes) to have ‘attractant’ properties, while hydrocarbons tend to be ‘repellent’ [98]. The final orientation step involves chemosensory cues at the root surface inducing acceptance or rejection. Root-associated microorganisms and co-occurring herbivores impact on these processes, equally affected by soil properties, whether they act on semiochemical diffusion or insect motility. Holistic approaches in realistic soil conditions should help determine the relative roles of CO_2_ and other volatiles as host location cues and how these relate to the host-plant range [90,98].

The use of natural, plant-based chemistry is gaining greater attention, due both to increases in analytical capability and the enhanced ability to dissect complex biochemical systems [40]. The soil volatiles are valuable infochemicals and will have to be considered to understand the entire integrity of the ecosystems. They constitute a new source of tools for belowground pest management [99,100]. Quantification of specific herbivore-induced released plant compounds and assessing their role in the increase or decrease of the attractiveness of a plant host to another type of herbivore will perhaps enhance our capability to manage populations of soil-dwelling herbivore pests and, thus, secure optimal outputs. Filling the current gap of knowledge concerning the role of belowground host plant location cues should enhance the improvement of IPM tools against subterranean insect pests [98], tools that would be very useful for wireworm control.

## 4. From Risk Assessment to Mass Trapping

Wireworms do not cause systematic damage during all the critical cropping phases, year after year. The first step in their management consists in assessing the risk of crop damage, *i.e.*, the level of populations in place, preferably in relation to crop sensitivity thresholds. For example, if early spring soil samplings indicate the presence of high levels of *A. sordidus* last instars, damage on sensitive crops sown during the same spring can be forecast; if high populations of younger larvae are found in June-July, severe attacks may occur in sensitive crops sown in late autumn or the following spring [41]. Crop rotation, availability of food resources through the season, climatic-agronomic features and soil characteristics are the main known factors influencing the composition of species communities and larval population density [57]. At the local scale, site-specific variables seem to show a positive association with the level of infestation, like grass duration (or surrounding grassy margins and weedy spots) [2,13,96,101,102,103]. The presence of meadows and double cropping within the rotation cycles would result in a population increase of species overwintering as adults [57]. However, for *A. ustulatus*, a negative correlation has been observed between spatial distribution of larval population and grass cover [104]. Another positive correlation is generally found with soil bulk density [13,96,101,103]. Correlations between soil humidity and infestation rate have also been reported [104,105]. 

But, the real difficulty lies in the mapping of zones where wireworm scouting should be performed preferentially because infestation is expected, combining time, space, agronomic and climatic variables [105]. The scale at which correlations are investigated is very important to define. In Hungary, when studied in small-scale maize fields and in a flat relief environment, *A. ustulatus* wireworm populations appeared clustered in 75% of cases [104]. A prediction model for wireworm activity and appearance in the damage zone, in relation to soil moisture, temperature and type, has been developed in Rhineland-Palatinate (Germany), with determination coefficients of 0.81 to 0.89 [89]. That very promising tool in risk assessment deserves to be tested in other regions. Multiscale correlations can be sought between landscape factors and damage levels on particular crops (which implies the defining of such levels). The distribution of wireworm damage in potato crops has been associated to landscape structure at a scale of 25 ha in Austria, grassy field margins being potentially the key landscape variable, sand content in the soil coming in second place (72% of the total variability explained by the two factors) [101]. Differences between *Agriotes* species distributions are closely linked to the scale and to species-specific factors. Predation, competition or social aggregation might intervene; the consideration of other pest/non-pest species in place is therefore essential [104,106], as when studying wireworm chemical ecology. Highlighting association/dissociation between species could help define indicator species useful for management. The impact of field or site characteristics may differ drastically between species. Also, the association between certain species might be linked to differences in soil pH, grass duration and the amount of organic matter content between fields. For so many reasons, there is a strong necessity to separate taxa into species when assessing the pest-complex [106]. Disregarding the distribution of the species forming the pest complex where integrated wireworm management is planned could serve for the modification of the pest species community without even affecting its size or impact on belowground stages of the pest. Developed below as risk assessment or mass trapping tools, the sampling methods we mention in this paper also relate to that necessity. 

### 4.1. Wireworms’ Sampling: From Prevention to Trap Crops

Several sampling methods for assessing infestation rate (number of wireworms per unit of surface) have been developed over time. The infestation thresholds above which treatments are recommended depend on the species, the proportion of voracious instars in the catches, the sampling method and the crops concerned [57]. Also, the limit of detection of traps must be smaller than the damaging population threshold [79]. Soil core sampling and counting of caught individuals lacks interest for growers and agronomists [79], because they are time-consuming [57,107] and subject to significant sampling errors, essentially due to the sampling effort, to the non-random aggregated distribution of the larvae [108,109] and particularly important in the case of low, yet damageable wireworm populations [110]. It is also important to consider that small larvae are more likely to be markedly aggregated, while medium-sized ones are more dispersed and large ones closer to random [109], a pattern involved in the interpretation of trap catches. Many bait-based sampling techniques have proven to be at least as effective as soil core for detecting wireworms [95,111,112]. Bait efficiency notably depends on the period of bait exposure (to be adapted according to case-specific parameters, such as trap handling or sowing period of the future crop) and the time of the year [28,112,113,114] and decreases when alternative food sources are present, which is often the case in freshly ploughed grass fields [24,79,115]. Germinating cereal seed baits, whose efficiency seems enhanced by daily irrigation [113], is the most efficient sampling technique (including cost and accuracy concerns) for determining wireworm populations in worldwide agricultural habitats and in grass fields intended for arable production [95,111,113,116,117]. 

Chabert and Blot (1992) used a modified version of traps designed originally against *Melanotus* spp. populations in the USA [118]. Briefly, they consisted of 650 ml plastic pots filled with vermiculite and a mix of 30 ml of maize and 30 ml of wheat as baits, entirely watered and buried in the ground, covered with soil and a plastic lid covered with a second soil layer. Traps were checked by hand after 10 to 15 days. For an equivalent number of samplings, baited traps allow the retrieval of two- to four-times more wireworms and save up to two thirds of the sampling time compared to simple soil core sampling [111,115]. On maize, the two techniques had comparable accuracies for high populations. When these decreased, accuracy remained acceptable with baited traps, while turning disastrous with soil cores. Such traps allowed linking the percentage of deceased plants to the wireworm infestation rate in several situations. For example, in maize, sowing before May 1^st^ in fields infested by five to 10 larvae per square meter caused the destruction of 30% of the plants [115]. Bait traps remain better presence/absence indicators of wireworm infestation than soil cores alone, especially where populations are below the limit of detection of soil cores (in potato – 62,500 wireworms/ha with 20 soil cores), even if the effective sampling area of a trap may be influenced by many factors [111,112]. In potato crops, there is a trend towards a higher level of damage in untreated plots with higher wireworm abundance, but even at densities of less than 100,000 larvae/ha, damage can range between 20% and 80% of tubers attacked [119]. Similar wide ranges were found in Romania: wireworm populations (mainly *Agriotes*) ranging from 5–10 larvae/sq.m to 70–100 larvae/sq.m will induce up to 31.6% of attacks on wheat, 22.5% on barley, 42.8% on corn seeds, 54.0% on corn at root neck, 64% on sunflower seed and 53.0% on sunflower at root neck [120]. This emphasizes the difficulty to define infestation thresholds above which treatment has to be applied and is partly attributable to sampling accuracy. A remarkable example of an efficient sampling plan to assess wireworm occurrence and control measure necessity was reported by Cherry *et al.* (2011). A sequential core-sampling plan at sugarcane planting was conducted on purpose to target areas needing treatment. If nine or more wireworms (*Melanotus communis* and *Conoderus* spp.) were retrieved from a total of 25 samples evenly spaced in a diagonal field transect, the latter were treated. By showing no significant differences in further yield and wireworm numbers between adjacent paired fields (identical agronomic conditions—one treated, the other untreated), they showed that both the sampling method and the used economic injury level allowed $100(US)/ha savings in unnecessary soil insecticide application [121]. In Italy, data collected over 15 years showed significant correlations between infestation rate or the average number of larvae per bait trap and damage in maize plants by *A. brevis*, *sordidus* and *ustulatus* [57]. Significant relationships between pre-planting catches of wireworms by baited traps and damage to maize plants were found for all three species, but symptoms of damage varied. When the population is greater than five larvae/trap, *A. ustulatus* larvae may significantly affect plant stands by damaging seeds. *Agriotes brevis* was found to be the most harmful, with catches above one per trap causing considerable plant damage that may result in a yield reduction. For the same damage level in maize fields, five-times more *A. ustulatus* larvae are needed. *Agriotes sordidus* has an intermediate damage potential (wireworm densities above two larvae/trap may cause a yield reduction). These thresholds are reliable for: (1) bare soil in which there are no alternative food sources, also after meadow (e.g., alfalfa and ryegrass) if this has previously been cultivated (in this case, the field has to have been ploughed at least three months before baited trap placement); (2) the average soil temperature at 10 cm depth is over 8 °C (for more than 10 days); and (3) the soil humidity is high (near to field water capacity) (Lorenzo Furlan—personal communication). These examples highlight the accuracy needed as to the conditions in which thresholds are described. 

It is generally admitted that further improvements in bait traps or other sampling methods will be needed to accurately estimate the density of wireworms. Considering the factors involved, the outcomes in terms of treatment decision baseline may be as various as existing agronomic situations, spatial distribution (large-scale patterns) and crop-wireworm species combinations. The combined use of soil-core sampling and bait trapping may give a more accurate representation of potentially damaging species present, because it reflects more properly the wireworm species and distribution within the soil [122]. The closer to reality the estimation of the population is, the more precisely damaging thresholds can be defined, therefore reducing unnecessary treatment cases. 

In-soil baits are useful in wireworm mass trapping, trap crops being the most efficient in that purpose, providing a very attractive alternative food source. By planting wheat rows eight days in advance of intercropped rows of strawberries, the seedling mortality due to *Agriotes* feeding can be reduced from 43% in unprotected plots to 5.3% in plots with an intercrop of wheat [5]. Maize protection can also be achieved and enhanced as the plant species diversity in the trap rows increases. Molecular analysis of gut content screening for plant DNA [86] outlines that wireworms are lured away from the target crop and also preferentially consume the intercrops [123], e.g., “trap peas” (*Pisum sativum* cv. Valverde) are more attractive to wireworms than potatoes [124]. These efficient techniques, difficult to set up at field scale, can only be developed with an economically interesting approach. 

### 4.2. Pheromone Trapping: Mass Trapping and Monitoring

Three main elements account for the fascination of insect sex pheromones and their feasibility for insect management: (1) they are species-specific (even when synthetic and incomplete, and blends usually affect only the target, with the possible exception of taxonomically closely related species); (2) they are active in very small amounts; and (3) the vast majority are not known to be toxic to animals [125]. The identification of click beetles sex-pheromone main compounds in the late 1980s provided the qualitative bases of sampling techniques and mating disruption/mass trapping according to species. 

Geranyl and (*E,E*)-farnesyl esters are major components of *Agriotes* natural sex pheromones [126]. Some species of click beetles may contain up to 24 substances in their pheromone glands [127]. Tóth (2012) summarized the status of identification of pheromones and attractants described in a variety of species among which 22 (73.3%) were *Agriotes* [128]. Click beetles show a variety of responses according to changes taking place in the structure of the pheromone component, especially in its acyl group or allylic fragment [129]. The sex attractant pheromone of one species may act as a sex inhibitor with respect to the other [129] through one of the compounds of the blend [130]. The same component can capture different target species, e.g., geranyl hexanoate actively attracts both *A. sordidus* and *A. rufipalis* Brullé [131]. Geranyl butyrate (GB) and (E,E)-farnesyl butyrate (FB) were identified in the pheromone gland extract of females *A. brevis* as the major sex pheromone components. Used as field baits (Italy, Hungary, Bulgaria) where both *A.sputator* and *A. brevis* were present, they selectively caught only *A. brevis,* whereas GB is also the main pheromone component of *A. sputator,* suggesting that FB has a role in reproductive isolation [132]. On the other hand, one sex-pheromone can attract individuals of species other than the target one, depending on geographic location [10,11,130,133], which shows that pheromone geographical “dialects” exist [9,127,129]. For *A. lineatus* and *A. proximus*, the comparative study between pheromone profile and the mitochondrial cytochrome c oxidase subunit I gene even led to the consideration of a necessary taxonomic revision [134].

The pheromone gland extract composition does not necessarily follow the proportions obtained by volatile collection when available, e.g., for *A. lineatus* and *A. proximus* [134], while the optimal ratio of concerned compounds in pheromone lures is also different from the two latter extracts [130]. In the future, if the pheromones of problematic species are to be analyzed by volatile collection (an unavailable method when most *Agriotes* gland extracts were studied in the 1980s), then more accurate pheromone elucidations may result. Besides, it has been noticed in some cases that females could be attracted towards blends of synthetic pheromonal compounds, revealing potential aggregation traits of the latter [128]. The same analytical improvements could provide information to understand these observations. 

Very recently, a binary combination of two synthetic floral compounds, (*E*)-anethol and (*E*)-cinnamaldehyde, was optimized as a female-targeted lure for female *Agriotes ustulatus* Schwartz click beetles, opening a new perspective for adult click beetle mass trapping [128,135], but most of the applied cases of adult monitoring have focused on male click beetles thanks to pheromone traps. Guidelines for the synthesis of sex-pheromone, highly effective for baits, are available [133,136]. Several sex pheromone traps have been developed for monitoring all the most important *Agriotes* species [9,137,138,139,140]. Among them, bottle traps (homemade funnel traps) and VARb funnel traps, an adaptation of CSALOMON Var funnel traps (Plant Protection Institute of Budapest, Hungary), were adapted for species privileging flying over crawling. TAL traps (adapted pitfall traps) and YATLOR traps, similar to the Estron trap described by Oleschenko (1987) and Kudryastev (1993) ([139] and [9]) were designed for species privileging crawling over flying. A combination of the bottom part of a YATLOR trap and the upper part resembling the bottle trap gave birth to a high capacity trap, the YATLORfunnel, or YATLORf trap, effective in monitoring all the target species during all the swarming season [137]. It allowed detecting wireworm population levels in different European countries [7] and establishing distribution maps of the predominant species, even at low population densities [10]. Trap design matters: during the early part of the trapping season, traps into which both crawling and flying beetles can enter may capture significantly more individuals than designs where insects can only fly in. This has been observed for *A. brevis* and disappears later in the season. This indicates the need for traps suitable for use throughout the whole season [132].

A ground-based pheromone trap highly efficient for monitoring *A. lineatus* and *A. obscurus* has also been developed in Canada (the “Vernon Beetle Trap”, Phero Tech Inc., Delta, British Columbia) [140], and attempts for their monitoring highlighted that pheromone trapping should be the choice method for their surveying in North America [141]. 

Blackshaw and Vernon (2006) demonstrated the usefulness of pheromone trapping as a sampling method in addressing ecological questions at a landscape scale, by studying the spatial stability in non-farmed habitats of male *A. lineatus* and *A. obscurus* [142]. Click beetle species have been monitored thanks to large-scale pheromone trapping in Croatia. The abundance and dominance of *A. lineatus* decreased compared to average data from 1961–1990, abundance being negatively correlated with average air temperature. This result was postulated to be a potential effect of climate change [12]. In Rhineland-Palatinate (Germany), pheromone trap catches performed from 2008 to 2010, with the aim to define click beetle communities, highlighted the unexpected presence of *A. sputator*, possibly related to the increased potato damage observed during the previous seven years [11]. Such studies perfectly illustrate large-scale direct uses of pheromone trapping. Interpreting adult male trap counts for quantitative predictions of population size is complex and limited, unless the observed differences between species can be explained by dissimilar movement rates of the species and interferences between traps [143], hence the necessity to acquire accurate knowledge of the range of attraction of the pheromone traps [144]. It is essential, although not necessarily always possible yet, to: (1) establish the biological significance of the pheromone trap catches; (2) determine the actual range of attractiveness (related to the movement rates—male *A. obscurus* are able to migrate at least 80 m and *A. sputator* up to 27 m, distances linked to site-specific factors [75,145]); (3) study the relationship between males captured and the level of the female population; and 4) establish a reliable correlation between adult trap catches and subsequent larval populations for all the species and varieties in different climatic and agronomic conditions (mainly rotation) [137]. In Italy, sex pheromone traps proved to be much more sensitive in detecting species than the tools used to monitor larval populations, and the trap catches were correlated with estimated larval populations [146]. A mark-release-recapture study performed in South Devon, U.K., in a topographically flat and wind obstacle-free area, helped in getting species-specific migration distance data. Inventories led to the definition of maximal sampling ranges and effective sampling areas of *A. lineatus*, *A. obscurus* and *A. sputator* traps. Recapture rates were significantly different between species and recapture distances (ranging from 4 to 32 m). With such results, an estimation of the minimum cost of mass trapping programs to prevent males from mating was possible: 165€/ha/year (*A.lineatus*), 247.5€/ha/year (*A.obscurus*) and 2343€/ha/year (*A.sputator*) [147], illustrating the importance of separating wireworms into species. Sufyan *et al.* (2011) worked on the same problem in Germany and obtained slight differences, mainly due to the employed regression method. They took account of the attractive potential, *i.e.*, the maximum sampling area of pheromone traps in the equation. On the basis of the estimated probabilities of recapture, a maximum distance of 20 m between individual traps would be needed to ensure substantial mass trapping, *i.e.*, 25 traps/ha to theoretically reduce the male click beetle populations by more than half [144]. 

The potential of using pheromone trapping for male click beetles with subsequent wireworm reduction remains unclear, but will definitely require a dense network of traps [144]. The spatial relationship between aboveground adults and belowground larval distributions may not always be straightforward, unlike for other pests, which is important in cases where the monitoring and/or management is carried out on a different life stage to that causing damage [122,148]. Unfortunately, the spatial relationships between adult male catches and the distribution of their larvae is such that pheromone traps alone will not reliably indicate where wireworms occur in a field or an area [143]. 

The use of sex-pheromones to monitor and trap *Agriotes* click beetles illustrates the issues separating the understanding of an ecological phenomenon at the individual scale and its application in IPM strategies. Behavioral and ecological differences between species need to be acknowledged when researching the pest complex at the field and larger scale [106,148,149]. Separating wireworms to species instead of grouping as a complex reveals more as to the relationship between adult and wireworm distributions [122]. Also, the usefulness of pheromone traps in assessing *Agriotes* spp. populations could be restricted to larger scales than those at which pest management is normally undertaken. Distribution maps should be defined using sex pheromone traps within zones defined according to prevalent crop rotation organic matter content, soil type and precipitation [57], as illustrated above. In fragmented rural landscapes, the monitoring of Elateridae should take into account the heterogeneous pattern of the adult population (stratified sampling) [150]. Combining a geostatistic analysis of agronomic characteristics of soil and climatic conditions of the investigated area with adult distribution pattern, it should be possible to draw risk-areas on a predetermined scale of investigation (such as a province) and to provide more precise monitoring and management, provided that further studies show more details of how key variables influence the distribution [150]. 

## 5. Cultural Manners

Beside avoidance of sensitive crops in highly infested areas [34,57], cultural manners efficient against wireworms can be summarized as follows: (1) avoidance of grass in the crop rotation, since it promotes oviposition, egg and larval survival and is generally correlated with further crop damage [2,34,75,77,78,102]; (2) end of spring drainage of slow soaking fields suitable for wireworm development; (3) appropriate crop choice in regard to the infestation rate (see above) and prolonged rotations with hooded crops in May to suppress suitable conditions for egg laying; and (4) soil tillage in late spring and late summer when larvae or eggs are in the upper soil layers to enhance their death by desiccation, mechanical injuries or predator exposure [34,57]. Agrobiocenozes characterized with intensive tillage may also affect click beetle abundance [13], potentially with population reduction year by year after grass cover [2]. Field flooding can be envisaged in infested fields, likely providing more effective control of wireworm populations in fall or summer (high temperatures), with an efficiency decreasing as soil salinity increases [151], but it requires significant field modifications [40].

In fields where the cropping history shows systematic infestation, a forward planning is required to allow the implementation of risk assessment methods well in advance of final decisions being made on field choice [79]. Preventing wireworm damage starts with avoiding growing particularly sensitive crops where and when infestation rates are significant (such as lettuce or maize highly sensitive to *A. sordidus* [41]). While non- or low-sensitive crops can be planted in infested fields, the remaining cultivated soil can be planted with any other crop [57]. Jary (1942) produced interesting guidelines to follow as to what crop should be preferred according to the level of infestation observed [77]. The sensitivity of crops to attack is highly variable [152] and the sensitivity of single seedlings and the one of the crop (agronomic sensitivity) should be distinguished [57]. The identification of crops and varieties less sensitive to wireworms than others is essential. It has been undertaken notably for potato varieties [6,92,153], sugarcane clones [3] or for different crops attacked by *A. ustulatus* [152]. Attacks on potatoes would be more important in organic farming systems [102]. Thresholds have to be defined for every existing situation (crop-wireworm species, method used to assess the infestation rate—see above), especially since *Agriotes* species show different responses to bait traps. For example, *A. ustulatus* can be three- to four-times more damageable to maize as to the number of damaged plants than *A. brevis*, which has an impact on the infestation threshold considered. The sowing period has to be considered equally. In controlled conditions, it is possible to define a precise scale of sensitivity. For example, two lettuce plants can be completely destroyed by 10–20 *A. ustulatus* wireworms, while there would be no plant loss for cabbage or capsicum in the same conditions [57]. 

Cultural manners could be combined with trap crop strategies undertaken preferably before any other treatment. Whenever impossible, the whole integrity of the systems should be revised, considering the economic issues involved, and point towards systems where they could be followed. 

## 6. From Broadcast Application of Synthetic Insecticides to Environmentally Friendly Alternatives

Extensive studies conducted in northern Italy outlined that less than 5% of fields planted with maize and sugar beet needed soil insecticides [154,155,156] or seed treatments [157]. Decisions to treat or not should rely on the threshold of infestation and not only on presence/absence indications [157]. Resorting to soil insecticide or insecticide-treated seeds to control wireworm populations is often unnecessary, but information to implement IPM strategies is missing or unknown to farmers [57]. When treatment has been shown unavoidable, efficient chemical alternatives to broadcast application of synthetic chemicals should be preferred. In this section, we discuss the alternatives and the importance of behavioral information brought to light by recent studies.

### 6.1. Seed Coatings with Synthetic Chemicals

Efficient active substances should be available in soil during critical cropping phases. Wireworms temporarily repelled or deterred from feeding on germinating seeds and seedlings will subsequently feed on older and less vulnerable tissue, which could be tolerated [21]. In any case, the timing of sowing in relation to upward wireworm movements in the root layer is a key factor to consider [158]. When corn is protected from damage during the first three weeks of growth, economic impacts can be minimized [40]. With seed coatings, the area treated with chemicals is considerably reduced and they appear just as effective as full-soil treatments. Moreover, they may well be combined with fungicide seed treatments, therefore providing easy handling by farmers together with adequate wireworm control, notably on wheat and barley [120,159]. Good candidates for seed coatings should unite four characteristics. They must (1) be efficient through wireworm feeding, *i.e.*, below integumental penetration rate insecticides, usually inefficient on wireworms [160,161], (2) be integrated in a solid state in the seed-coating mixture in order to avoid atmospheric dispersion during mechanical sowing, (3) have a low water solubility and a high affinity for soil particles and (4) remain active against wireworms after sowing (approximately one month for corn), during the whole seedling establishment/strong wireworm feeding phase [161]. Neonicotinoid insecticides as a treatment applied to potato seed at planting can enhance a significant reduction of attacked tubers between sowing and harvest (imidacloprid-70 g/ton seed, thiamethoxam-50 g/ton seed). Compared with broadcast organophosphorous soil treatments, the amount of needed insecticide can be reduced by over 90% depending on the amount of potato seeds per hectare. Such numbers, altogether with the lack of an adverse effect on potato yield, stand for an adequate alternative to soil treatments with organophosphorous compounds [159]. 

Seed treatments cannot automatically be linked with wireworm population mortality. On wheat, neonicotinoid (imidacloprid, clothianidin and thiamethoxam), pyrethroid (tefluthrin) and a combination of the latter and thiamethoxam applied as seed treatments can provide excellent stand protection, likely through prolonged wireworm intoxication, but the reductions of neonate and in place wireworm populations are not significant. To the contrary, fipronil seed treatment provides an excellent protection with population reduction, which makes it as efficient as lindane [162]. If blended with thiamethoxam, wheat stand and yield are improved over the individual chemicals applied alone. They lead to high mortality of resident and neonate wireworms, which implies this treatment should only be applied once every three years during which time susceptible crops could be grown, with much lower rates (50×) than the formerly used Vitavax Dual containing lindane [163]. Furthermore, if using seed treatment on only a fraction of the seeds, the proportion of treated seeds could even be adjusted to the infestation level: decreased proportions of treated seeds are needed when higher levels of larval infestation are observed, reducing even more the used quantity of active ingredients (0.095 g/ha). Such mixtures of untreated and blend-treated seeds could be used in push-pull strategies as trap crops [163]. This rate of application is as far as we know the lowest ever envisaged for wireworm control.

### 6.2. Natural Plant-Derived Chemicals

Control with naturally occurring active substances is possible. Both mortality and morbidity depend on the target species, and the specific effects should be taken into account [164]. Besides, if a biopesticide, intended as a plant-derived component, has a beneficial effect on the control of a pest species, potential phytotoxic effects on specific crops must be tested before any field trial [40].

The bioactive hydrolysis products of glucosinolates (GLs - contained in several *Brassica* species), particularly isothiocyanates (ITC), can be used to control soil pests and weeds through in-soil incorporation of GLs-containing plant material, a practice known as biofumigation. The relatively rapid sorption and degradation of ITC in the days after incorporation minimizes its persistence and leaching risks, which makes it a promising technique in the aim to reduce reliance on synthetic pesticides [165]. Allyl-isothiocyanate (AITC) has been tested on several important wireworm species. To our knowledge, Tattersfield and Roberts (1920) were the first to show its high respiratory-toxic effect on click beetle larvae, among a wide range of other substances [166]. Laboratory assays led to the assessment of the AITC LC_50 _for *Limonius californicus* and showed its lethal and sublethal effects, which in the meantime highlighted the potential of that product for other wireworm species control [21]. The key factor of efficiency is the GL richness of the plant tissue or derived meal used for control [167]. Biocidal plant root systems (*Brassica juncea* var. ISCI 99) do not necessarily cause significant mortality, while the incorporation of aboveground material at a dosage of 55 t/ha of fresh matter, corresponding to about 290 µmoles of GLs/l of soil, can significantly reduce wireworm populations [78]. In both pot assays and field trials for *Agriotes brevis*, *A. sordidus* and *A. ustulatus* management*,* the AITC tested sources were chopped *Brassica juncea* (L.) Czern fresh plants and biofumigant meals derived from defatted seeds of *Brassica carinata* A. Braun. In pot assays, a clear rate effect was demonstrated, with sufficient seed meal to supply approximately 160 µmoles of GLs/l of soil, resulting in significant wireworm mortality. The effect of the chopped *B. juncea* plants was less consistent. Accurate incorporation of defatted seed meals resulted in an efficacy level sufficient to protect susceptible crops from damage. Globally, the level of crop protection was comparable to that of conventional insecticide treatments and no evidence of phytotoxicity was noticed [168]. Spotting the appropriate timing for broadcast application is the critical factor here, because the active volatile substances remain available for only 24–36 hours [169]. Successful practical results depend on simultaneously united conditions: (1) suitable GL dosage (the target dose is related to infestation rate and degradation parameters); (2) homogeneous broadcast of biofumigant defatted seed meals, (3) effective and prompt soil incorporation, (4) suitable soil temperature (10.5–16 °C, available in spring and autumn in most European countries) and sufficient soil water content (enzymatic hydrolysis of glucosinolates) and (5) the occurrence of wireworms in the upper soil layers [168,169]. To ensure this, 10–15 days with a soil temperature over 18–20 °C after cold or dry conditions should pass so that the larvae in the little mobile stage in the deeper soil layers can turn into the feeding stage and move upwards looking for food [169]. It may also be possible to improve population management by applying biofumigant defatted seed meals when the first instar larvae are present, which would induce significant reduction in the following year’s population [168,169]. 

A few other plant-derived active substances affect wireworms. Cinnamaldehyde can significantly reduce damage to mother tubers when applied as a drench at 150 g a.i./ton tubers [170]. The most effective active ingredient in the neem tree *Azadirachta indica* A. Juss insecticides is azadirachtin. Its repellent effect on the sugarcane wireworm *Melanotus communis* decreases overtime. Future research determining whether repellency at different rates may be useful in reducing wireworm damage in both sugarcane and other agroecosystems and on other wireworm species [20]. Naturally occurring monoterpenoid essential oil constituents thymol, citronellal, eugenol and rosemary essential oil have been tested for their acute toxicity (LD_50_ and LC_50_) against late instars of *A. obscurus.* Their phytotoxicity was evaluated on corn germination and seedling establishment. Thymol had the greatest contact toxicity, while rosemary oil did not show any. Citronellal had the greatest volatile toxicity, before rosemary oil, thymol and eugenol. Thymol, eugenol and citronellal had significant phytotoxic effects (inhibition of corn seed germination and seedling establishment) and rosemary had only minimal ones. Acceptable qualities of a biopesticide therefore include direct toxicity and repellency effects on the pest. Also, for economic reasons, the best way to use these compounds would be according to their repellency and not their toxicity, therefore, in push-pull strategies [40]. 

The efficacy of tefluthrin in wheat seed treatments is likely due to a combination of repulsion and short-term morbidity events [162], but although repellency to an insecticide may provide temporary protection to plants and allow stand establishment, wireworms initially repelled may damage crops later in the season. Thus, it is important to observe the direct effects of novel insecticides on them. Chemicals identified as eliciting repellency should be tested to determine whether the attractive cues of host plants dominate repellent stimuli [171]. Moreover, the ability of wireworms to recover from an active substance-induced morbidity may seriously limit the efficacy of the substance, as it has been shown for *L. canus* and tefluthrin-treated seeds [172]. Their sensitivity to repellent compounds may decrease when repeatedly made moribund. They may even be capable of associative learning [23]. Whenever they are conducted, toxicity trials should include observations on morbidity, and the trials should last until morbidity symptoms cease. Long-term morbidity (lethal, sublethal and behavioral effects) and potential recovery or death of wireworms exposed to certain insecticides have implications on how laboratory and field studies should be designed and interpreted [30,162]. The on-going work on alternative active substances that affect wireworms can benefit lessons from previous research on synthetic chemicals. Table 1 shows insecticides tested against *Agriotes* wireworms since 1990. Some studies in which identification to the genus wasn’t stated were cited for their impact on research against wireworms. We distinguish between studies with behavioral observations on the larvae in regard to the insecticidal effect and those with field efficiency and crop protection considerations alone. Most of the behavioral effects (repellency, morbidity, sublethal effect, *etc*.,) concern *A. obscurus* and should be conducted on other species. Wireworm mobility categories proposed by van Herk and Vernon (2011) should allow comparing active substance efficiencies and species resistance [173]. Side effects on non-target organisms and acute or chronic effects on human health will always have to be investigated [40,169]. For any substance, field-realistic doses should be tested on potentially armed organisms, such as studied on bees for imidacloprid and clothianidin [174]. Globally, considering the overall apparent recovery and avoidance abilities of wireworms, push-pull strategies could be an interesting approach, with the advantage of limiting natural selection [40,175].

**Table 1:** Insecticides tested against *Agriotes* since 1990, with sources, mode of application, protected crop, *Agriotes* species (whenever possible) and specific behavioral observations (sublethal effects, such as morbidity, lowered activity and recovery periods or repellency).

Active Substance	Source	Application Mode	Target Crop	*Agriotes* species	Behavioral Observations
**Organochlorines**					
Lindane	[176]	Granular, Furrow treatment	Corn	*A. lineatus*	
	[177]	Furrow treatment	Corn	*A. lineatus,* *A. segetum*	
	[120]	Seed dressing (+ fungicide)	Wheat, Barley	*Agriotes* spp*.*	
	[178]	Seed dressing	Wheat	*A. obscurus*	
	[29]	Seed dressing	Wheat	*A. obscurus*	x
	[179], [171], [30]	Soil amendment/Topical application, Soil less bioassay, Dermal exposure		*A. obscurus*	x,x,x
	[162]	Seed dressing	Wheat	*A. lineatus,* *A. obscurus*	
Metoxychlor	[178]	Seed dressing	Wheat	*A. obscurus*	
Aldrin	[38]	Broadcast	Potato	*Agriotes* spp*.*	
**Organophosphates**					
Chlorpyrifos	[164]	Soil amendment/Topical application		*A. obscurus, A. sputator*	x
	[170]	Furrow treatment	Potato	*Agriotes* spp*.*	
	[159]	Furrow treatment	Potato	*A. lineatus,* *A. obscurus*	
	[179]	Soil amendment/Topical application		*A. obscurus*	x
	[171]	Soil less bioassay		*A. obscurus*	x
	[177]	Furrow treatment, Granular	Corn	*A. lineatus,* *A. segetum*	
	[30]	Dermal exposure		*A. obscurus, A. sputator*	x
Diazinon	[178]	Seed dressing	Wheat	*A. obscurus*	
	[179]	Soil amendment/Topical application		*A. obscurus*	x
Ethoprophos	[38],	Broadcast	Potato	*Agriotes* spp*.*	
	[159]	Broadcast	Potato	*A. lineatus,* *A. obscurus*	
	[170]	Granular, Broadcast	Potato	*Agriotes* spp*.*	
	[180]in [79]		Potato		
Fonofos	[38]	Broadcast	Potato	*Agriotes* spp*.*	
**Carbamates **					
Aldicarb	[38]	Broadcast	Potato	*Agriotes* spp*.*	
Carbofuran	[38]	Broadcast	Potato	*Agriotes* spp*.*	
	[177]	Furrow treatment	Maize	*A. lineatus, A. segetum*	
	[176]	Granular, Furrow treatment	Maize	*A. lineatus*	
	[120]	Seed treatment	Sunflower	*Agriotes* spp.	
Carbodan	[120]	Seed treatment	Sunflower	*Agriotes* spp.	
Thiofanox	[38]	Broadcast	Potato	*Agriotes* spp*.*	
**Neonicotinoids**					
Imidacloprid	[181]	Seed dressing	Corn	*A. lineatus*	
	[182]	Seed dressing	Corn	*Agriotes* spp.	
	[183]	Seed dressing	Wheat	*Agriotes* spp.	
	[158]	Seed dressing	Sugar beet	*A. brevis,* *A. ustulatus*	
	[184]	Seed dressing	Sugar beet	*Agriotes* spp.	
	[170]	Seed dressing	Potato	*Agriotes* spp*.*	
	[159]	Seed dressing	Potato	*A. lineatus,* *A. obscurus*	
	[179]	Soil amendment/Topical application		*A. obscurus*	x
	[171]	Soil less bioassay		*A. obscurus*	x
	[30]	Dermal exposure		*A. obscurus*	x
	[162]	Seed dressing	Wheat	*A. lineatus,* *A. obscurus*	
	[120]	Seed dressing	Sunflower	*Agriotes* spp.	
	[185] in [79]				
Acetamiprid	[179]	Soil amendment/Topical application		*A. obscurus*	x
	[120]	Seed dressing		*Agriotes* spp.	
Clothianidin	[164]	Soil amendment/Topical application		*A. obscurus,* *A. sputator*	x
	[179]	Soil amendment/Topical application		*A. obscurus*	x
	[171]	Soil less bioassay		*A. obscurus*	x
	[30]	Dermal exposure		*A. obscurus,* *A. sputator*	x
	[162]	Seed dressing	Wheat	*A. lineatus,* *A. obscurus*	
Thiacloprid	[159]	Seed dressing	Potato	*A. lineatus,* *A. obscurus*	
Thiamethoxam	[179]	Soil amendment/Topical application		*A. obscurus*	x
	[120]	Seed dressing	Sunflower	*Agriotes* spp*.*	
	[162], [163]	Seed dressing	Wheat	*A. lineatus,* *A. obscurus*	
	[159]	Seed dressing	Potato	*A. lineatus,* *A. obscurus*	
	[186]	Seed dressing	Tobacco	*A. ustulatus*	
	[182]	Seed dressing	Corn	*Agriotes* spp.	
	[187] in [79]				
**Pyrethroids**					
Bifenthrin	[188], [173]	Soil amendment/Topical application	Potato	*A. obscurus*	x,x
	[120]	Seed dressing	Sunflower	*Agriotes* spp.	
Cyfluthrin	[180]in [79]		*Potato*		
Tefluthrin	[29]	Seed dressing	Wheat	*A. obscurus*	x
	[179]	Soil amendment/Topical application		*A. obscurus*	x,x
	[171]	Soil less bioassay		*A. obscurus*	x
	[30]	Dermal exposure		*A. obscurus*	x
	[120]	Seed dressing	Sunflower	*Agriotes* spp.	
	[162]	Seed dressing	Wheat	*A. lineatus,* *A. obscurus*	
	[186]	Seed dressing	Tobacco	*A. ustulatus*	
	[182]	Seed dressing	Corn	*Agriotes* spp.	
**Phenyl pyrazole**					
Fipronil	[160], [161]	Feeding assay, Seed dressing	Corn	*Agriotes* spp*.*	x,x
	[179]	Soil amendment/Topical application		*A. obscurus*	x
	[30]	Dermal exposure		*A. obscurus*	x
	[162]	Seed dressing	Wheat	*A. lineatus,* *A. obscurus*	
	[163]	Seed dressing	Wheat	*A. obscurus*	
	[158]	Seed dressing	Sugar beet	*A. brevis,* *A. ustulatus*	
	[168]	Seed dressing	Corn, Lettuce	*A. sordidus,* *A. brevis,* *A. ustulatus*	
	[120]	Seed dressing	Sunflower	*Agriotes* spp.	
	[189]in [79]		Potato		
**Spinosyn insecticides**					
Spinosad	[190]	Soil amendment/Topical application		*A. lineatus,* *A. obscurus*	x
	[179]	Soil amendment/Topical application		*A. obscurus*	x
**Nematicides**					
1,3-dichloropropene	[191]	Soil amendment/Topical application		*Agriotes* spp*.*	x
Fosthiazate	[191]	Granular		*Agriotes* spp*.*	x
**Fungicides**					
Difenoconazole and Mefenoxam (Dividend XLRTA)	[29]	Seed dressing	Wheat	*A. obscurus*	x

## 7. Biological Control Using Micro- and Macro-organisms

### 7.1. Entomopathogenic Fungi

The efforts to develop the use of entomopathogenic fungi (EPF) against wireworms are very recent, although these enemies were known since early in the 20th century. To our knowledge, the first report of *Metarhizium anisopliae* Sorokin as able to infect wireworms in nature was made in 1932 [192]. It can be applied in the field and infect and kill wireworms [193]. Laboratory assays allowed identification of virulent strains of EPF against larvae of *A. lineatus*, namely V1002 and LRC181A, respectively able to cause 90 and 100% mortality three weeks post-inoculation [194]. Applied before planting in an area with wireworms (95% *A. obscurus*) as a corn seed treatment alone or in combination with clothianidin or spinosad, the strain F52 can induce significant increases in stand density and stock and foliage area fresh weight yield. Not only does *M. anisopliae* strain F52 growth observed on retrieved wireworm cadavers link the positive effects on corn with the wireworm control, but it also suggests a synergistic interaction between EPF and insecticides [195], like when the strain is combined with spinosad: *A. obscurus* and *A. lineatus* wireworm mortality is higher when treatments are combined than for each treatment applied alone [190]. The efficacy of *M. anisopliae* can also be enhanced by incorporation of neem seed cakes, as shown for the control of the black vine weevil *Otiorhynchus sulcatus* (Coleoptera: Curculionidae) [196]. Moreover, because *M. anisopliae* gradually degrades in the soil, the inclusion of spinosad or other insecticide treatments could extend the total control period provided by one application [190]. 

*Beauveria bassiana* (Bals.-Criv.) Vuill. is also efficient, even more with low doses of imidacloprid [170]. Presumably, the low rate (bio)insecticides stress the target or alter their behavior and immune mechanisms in such a way as to make them more susceptible to infection [190,197,198]. Some insecticides may increase pest mobility and increase acquisition of EPF conidia and, since mortality is dose-related, increase mortality [198]. Low levels of reduced-risk pesticides can be combined with biological agents without environmentally harmful traditional pesticide strategies [194,198], which clearly represents potential savings for growers [198]. 

Further investigations concerning virulent strains of EPF should be undertaken taking into account factors, including temperature, duration of exposure to the studied strain, soil concentration of conidia and food availability. These will affect the levels of mortality and the rate of emigration of wireworms repelled by the fungi [199]. The use of fungus against click beetles is less developed, though *Zoophthora elateridiphaga* has been found to be an important mortality factor of the click beetle *A. sputator* in Swiss meadows [200] and strains efficient against larvae have been suggested as potentially equally efficient against click beetles [194].

### 7.2. Entomopathogenic Nematodes

Entomopathogenic nematodes (EPN) are also efficient against wireworms. *Steinernema feltiae* and *S. glaseri* caused a high mortality on the sugar beet wireworm *Limonius californicus*, however, field experimentations did not show very efficient and economically interesting results. The timing of application in this regard is a very important factor [201]. On *Agriotes* wireworms, *S. feltiae* furrow treatments have been tested, but were not efficient [170]. The strain UWS1 of *Heterorhabditis bacteriophora* Poinar has proven very efficient against *A. lineatus* (67% mortality three weeks post-inoculation) [194]. *Hexamermis* spp. is also a known parasite of *A. obscurus* [202]. Field damage to maize by wireworm can significantly be reduced with applications of *H. bacteriophora* and *Steinernema* species [203]. The most efficient strains among those screened by Ansari *et al.* (2009) were local and originated directly from wireworm cadavers [194]. Where IPM is considered, native strains that co-evoluted with a target pest, and therefore adapted to local conditions, should be screened for their efficiency. More than identifying perfectly adapted strains less risky as to their impact on non-target organisms, this helps outline their optimal development conditions. This was undertaken for Spanish strains of *S. feltiae,* even if, in this case, the most efficient one only led to a 7% larval mortality on *A. sordidus* [204]. Any potential remains interesting because of possible synergies [198], such as observed with *M. anisopliae* in the control of the pest *Hoplia philanthus* Fuessly (Coleoptera: Scarabaeidae) and the black vine weevil *O. sulcatus* [205,206]. Synergies could equally work against wireworms and offer an organic or chemical-free approach in the control of *A. lineatus* [194] and potentially other *Agriotes* species.

The mechanisms underpinning synergy between agents remain unclear. Furthermore, not all interactions are synergistic. Antagonistic or barely additive effects have been observed in a wide range of situations involving other pests, strains and pesticides. The outcomes appear to be dependent on factors, including EPF strain (a virulent strain is vitally important), dose and the synergist type or efficacy enhancing agent used, which overall underlines the need to identify and optimize synergies [198]. Differences in wireworm species susceptibility to EPN exist and may be due to a range of physical barriers, including morphological barriers or behavioral responses to attacks [207]. Once more, this highlights the necessity to consider the overall pest complex case by case.

Comparisons between existing management options is necessary, whether they concern different treatments or consideration of potential control agents in place. The outcomes of such comparisons may differ drastically between agronomic situations. For example, natural infestations of *Metarhizium* spp., *Beauveria* spp. and nematodes should be evaluated when studying any treatment, as was done for biocidal seed meals in a field trial with rearing cages (*A. sordidus* and *A. ustulatus*) in different crop rotation systems. In this case, their efficiency was overall low and inferior to that of GL-containing plant material and did not differ between rotations [78], but one can imagine the biases caused by omitting their roles in the results interpretation. 

### 7.3. Other Organisms

Searching among dead wireworms collected in Germany in the aim to find new entomopathogenic taxa, an intracellular bacterium was recently identified: a pathogen belonging to the genus *Rickettsiella* [208,209]. Its suitability for IPM strategies hasn’t been tested yet. The insecticidal potential of the bacterial flora of *A. lineatus* and other hosts was also studied. Of the isolated strains, five induced 100% mortality on third instar *A. lineatus* larvae 10 days after treatment, three of them belonging to the wireworm flora, highlighting their microbial control agents’ potential [210]. Among potentially interesting predators of wireworms, some Carabidae and Staphylinidae (Coleoptera) are known to feed on *A. sputator* [211], but their use as control agents is inexistent. Other predators or parasitic pathogens should be researched, for example, in systems where everything points to wireworm infestations without occurrence. Then again, such natural control is possible in very diversified agricultural systems, where there is room for species other than crops and their pests. As stated above, grassy landscape margins would be key factors explaining wireworm potato damage. Their role for the maintenance of natural pest control should be investigated [101], especially since weed spots are key landscape elements for oviposition [103].

## 8. Conclusions

According to Furlan (2005), a rational IPM strategy against wireworms should be based on 1) locating high risk areas, notably with sex pheromone traps, 2) planting sensitive crops in areas with very low or no risk and 3) locating areas with actual *Agriotes* populations over thresholds. If no economic larval population is present, sensitive crops may be sown without any treatment. Where economic populations have been found, sensitive crops should be sown late in the season or the next year, after application of an adequate treatment (biological or insecticide, period, rate) to control larval stages present, and tillage should be undertaken in the most suitable period to ensure high mortality [57]. In infested fields, damage may not occur every year and can be forecast by detecting wireworms at those periods, to prevent from further damage [41], provided that the lifecycles of the species in place are well known and that mortality is ensured by the technique resorted to in the problematic areas. 

As outlined throughout this paper, rational IPM strategies are available, and the treatment options or monitoring tools in this regard are in good development. Whenever considered, any element of IPM strategies should be planned, taking into account the encountered wireworm species, because of the differences in biology, ecology and behavior that can occur between them. Species for which gaps of knowledge remain should be studied following a similar methodology to those already well described [57]. Although not conceivable for growers, the identification of the main *Agriotes* pest species is possible, thanks to morphological characteristics or the genetic footprint [8,49,50,55], a tool that will probably, in the near future, outline more the diversity of pest populations in place [58]. Wireworm sampling is time-consuming and not always reliable. While there are examples of methods efficient for infestation/attack rate assessment (maize, France and Italy [57,115]) or treatment decisions (sugarcane, Florida [121]), counter-examples exist (potato [79,110,111,112,119]). When the level of infestation can’t be related to adult trapping, the latter could be used as a control tool, since the components of the main *Agriotes* species sex pheromones have been identified and proportions optimized for efficient baits [126,128,129,133]. Their potential in wireworm reduction over the years following male click beetle mass trapping remains unclear and may require a dense network of traps [144]. More research should focus on the comparison of agricultural systems: those where attacks are nearly systematic and devastating compared to those where attacks are rare or still not intense enough to reduce yields significantly. Enough data should be acquired on both types of situations so that large-scale analysis can help point out the subtle combination of factors that remain invisible for now [96]. The impact of field or site characteristics may differ drastically between species, hence the strong necessity to separate taxa into species when assessing the pest-complex [106]. This could highlight more of what should be considered a priority when managing wireworms in a preventive way. Chances are that the most efficient IPM strategies will take place in the most diversified agricultural systems. A preventive systemic approach is always the most interesting to consider on the long-term. Moreover, it gives time for treatment options to be developed and repeatedly improved.

The resort to insecticides can be much reduced, notably by privileging seed treatment, which can ensure seedling establishment where and when it is the most needed [159,162,163]. Besides, when insecticides are combined to other control agents, such as entomopathogenic fungi or nematodes, synergies may be observed [190,194,198]. Promising studies highlighted the effects of natural substances-derived chemicals on wireworms and their potential as synergists as well (e.g., [20,168,190,195,196,198]). They should be preferred to insecticides whenever possible, especially regarding the latest European regulations in the matter of plant protection and their implications [212]. Among such substances, those ensuring mortality have effects lasting longer than stand establishment, which is economically more relevant, but considering the overall apparent recovery and avoidance abilities of wireworms, push-pull strategies could be an interesting approach, with the advantage of limiting natural selection of the pest [40,175]. In order to compare and identify what synergies are the most reliable on wireworms, potential synergists and other available tools should be reunited in common trials and their combinations tested on several, if not all, of the most important *Agriotes* species, through a standard methodology developed for different regions. Given the concerns of environmental effects, such as animal and human health, any product destined to control wireworm populations should always be studied as to its potential side effects with field realistic doses (to be established in every case), such as investigated for the effect of neonicotinoid seed treatment residues on honey bees [174].

Regardless of treatment efficacy, there are differences in the susceptibility for wireworm damage between crops, cultivars and farming systems [3,6,41,57,77,92,102,152,153]. The production of more resistant cultivars thanks to breeding may rely on the understanding of mechanisms underpinning these differences. Improvements of current knowledge on wireworm chemical ecology could help in achieving that purpose. Johnson and Gregory (2006) suggested that other plant-produced products other than CO_2_ have a role in the feeding choice of the larvae, notably root-emitted volatiles [90]. The identification of such semiochemicals might help explain the diverse sensitivity among varieties. Studies on specificity and variability of belowground responses should be included in efforts to exploit tritrophic interactions to improve biological control practices [213]. Such advances could lead to both varietal selection and new integrated wireworm management options.
